# Working Conditions and Self-Reported Health Among Undocumented and Newly Regularized Migrants in Geneva: A Cross-Sectional Study

**DOI:** 10.3389/ijph.2023.1606394

**Published:** 2023-12-06

**Authors:** Munire Hagose, Claudine Burton-Jeangros, Julien Fakhoury, Liala Consoli, Jan-Erik Refle, Yves Jackson

**Affiliations:** ^1^ Faculté des Sciences de la Société, Université de Genève, Geneva, Switzerland; ^2^ LIVES Swiss Centre of Expertise in Life Course Research, Geneva, Switzerland; ^3^ Centre Interfacultaire de Gérontologie et d’Études des Vulnérabilités, Université de Genève, Carouge, Switzerland; ^4^ Division de Médecine de Premier Recours, Hôpitaux Universitaires de Genève (HUG), Geneva, Switzerland

**Keywords:** migrants, occupational health, undocumented, regularization, self-rated health

## Abstract

**Objectives:** Most undocumented migrants are employed in so-called “dirty jobs” or “3D jobs” (dangerous, dirty and degrading) due to their lack of legal status. This study aimed to describe the self-reported health of undocumented and newly regularized migrants in relation to their working conditions.

**Methods:** A cross-sectional study was conducted using data collected during the first phase of the *Parchemins* study (2017–18), a survey that monitors the socioeconomic and health impact of a regularization scheme for undocumented workers in Geneva, Switzerland. The sample consists of 395 undocumented and newly regularized migrants.

**Results:** Overall, 147 (37.2%) rated their health as very good or excellent. Multivariable regression analysis indicated that work-related factors associated with better self-reported health included higher satisfaction with working conditions, while legal status regularization showed only a borderline association. By contrast, workers performing very demanding tasks and having more difficulties finding a new job were less likely to report very good or excellent health.

**Conclusion:** Findings show that work-related factors had a stronger influence on self-reported health compared to legal status change. Further research is needed to evaluate the long-term impact of regularization on working conditions and self-rated health.

## Introduction

International migration is increasing globally. In 2020, the number of migrants was estimated to be 281 million, which represents 3.6% of the world’s population [1]. As the term migrant worker is used to define underlying motivations for migration or refers to the legal or employment status of the migrant, it is difficult to precisely determine the size of this specific segment of the migrant population [2]. The International Labour Organization (ILO) defines migrant workers as people living in a country other than their country of origin, who are working or seeking work in the host country or were previously seeking work or employment. ILO estimates that approximately two-thirds of the migrant population are migrant workers [3]. These workers significantly contribute to global social and economic development, not only to the economy of their country of origin via remittances, but also by providing a labor force and various levels of skills to the host country [4, 5]. The decision to migrate is supported by the aspiration to improve current living and economic conditions, as well as those of relatives, by engaging in income-generating activities outside of the country of origin [6, 7]. Migrant workers lacking a valid residence permit in the host country are defined as undocumented. They constitute a highly vulnerable population given their lack of social and legal protection and their exposure to cumulative forms of disadvantages [8].

Previous studies have shown that precarious living conditions place undocumented migrants at risk of negative health outcomes [4, 5, 9], which is reflected in the frequent and early occurrence of mental health issues and chronic conditions in this population [9–12]. The lack of legal status makes migrant workers vulnerable to coercion in numerous domains, including work [6, 13]. Indeed, undocumented migrants are more likely to find themselves in conditions of employment that are prejudicial to their health, such as servitude, forced labor, human trafficking or slavery [6, 14]. In addition, this population is often reluctant to denounce its dangerous and precarious living and working conditions through fear of denunciation and deportation [8, 15, 16].

The majority of undocumented migrant workers are employed in so-called dirty jobs [also known as 3D (dangerous, dirty and degrading) jobs], in the hospitality, construction or domestic and care industries [13, 14, 17]. These workers often do not benefit from any control over their work conditions while they are employed in low paid jobs that generate significant physical and emotional strain [18, 19]. Links between precarious working conditions and adverse health outcomes have been previously described in documented migrant workers [8, 20, 21]. For instance, a high prevalence of osteoarticular pain related to physical activity, work intensity and ergonomic constraints was observed among domestic workers [22], frequent respiratory illnesses were reported among migrants employed as cleaners [23]. Precarious working conditions were associated with psychological distress [24] and job insecurity may have adverse effects on somatic and psychological health for migrant workers [25]. In addition, irregular employment often results in a high incidence of chronic health problem problems [12]. Hence, this evidence supports the hypothesis that the absence of legal status may affect the health of migrant workers.

However, research specifically focusing on self-reported health among undocumented migrant workers is scarce for different reasons. First, the topic has traditionally triggered limited interest from researchers and research-funding agencies. Second, this population often remains invisible in the public and social arenas, as the result of protective strategies against identification and deportation, which makes it hard to reach for researchers [26]. Third, there are complex methodological challenges to overcome to study the interplay of factors involved in a longitudinal perspective on regularization [27].

Legal status regularization may theoretically create better working conditions. Indeed, it can operate at different levels including the ability of workers to seek employment in the formal job market and in better regulated sectors, applying for jobs that better match their previous qualifications, and gaining additional bargaining power with the employer, among other benefits. Regularization may therefore be associated with a better health status as measured by objective indicators and a better assessment of one’s health using subjective evaluation tools. To the best of our knowledge, no study has formally assessed this relationship in Europe. To address this knowledge gap, the present study aims at describing the self-reported health of undocumented migrants in relation to their working conditions and at assessing the relationship of legal status regularization with their self-reported health in a Swiss setting.

## Methods

### Study Design and Participants

This cross-sectional study reports baseline data collected between 2017 and 2018 as part of the prospective, multidisciplinary, mixed-methods *Parchemins* study, which aims at monitoring the impact of a conditional regularization program on undocumented migrants’ health and living conditions in Geneva, Switzerland (anonymized).

Switzerland is host to between 58,000 and 105,000 undocumented migrants, of which 10,000 to 15,000 live in the Geneva area [19, 28]. In 2017, the State of Geneva launched a pilot scheme called “Operation Papyrus” with the goal to decrease the number of undeclared workers and regularize the residence status of undocumented migrants meeting strict criteria [29]. Candidates had to meet the following five eligibility criteria: not being registered as an asylum seeker; proving continuous residence in Geneva for the past 10 years (5 years for a family with a school-age child); be financially independent (without social assistance); have no criminal record; and possess a basic level of French language proficiency. Through this scheme, 2,390 undocumented migrants were regularized [29].

The multidisciplinary and mixed-methods *Parchemins* study was initiated in parallel to the implementation of the “Papyrus” scheme in order to monitor its impact on the health and living conditions of migrants (anonymized). Inclusion criteria to the *Parchemins* study were: age >18 years; citizenship of a country outside of the European Union (EU) or the European Free Trade Association (EFTA); a duration of stay in Geneva of at least 3 years without a residence permit; plans to stay in Geneva for at least three additional years; and no previous registration as an asylum seeker. Participant recruitment took place in various locations in the community and in a dedicated unit of the Geneva University Hospitals, which provides primary care to undocumented migrants. Trade unions and associations in contact with this population also helped to recruit participants for the study. A full description of the recruitment strategy is provided in (anonymized).

### Data Source

Investigators administered a standardized face-to-face questionnaire available in the four most common languages within this Geneva migrant community (English, French, Portuguese and Spanish) according to the participant’s preference (anonymized). Responses were recorded in a mobile device and transferred to a secure server located at the University of Geneva. Interviews took place either at the University of Geneva or at locations chosen by the participant. All participants provided written informed consent. The study protocol was approved by the ethics committee of the Canton of Geneva (CCER 2017–00897).

#### Dependent Variables

The dependent variable was self-rated health (SRH), an easy-to-collect, valid and frequently used indicator in epidemiological studies of physical and mental morbidity and a predictor of mortality [30, 31]. SRH was measured using question 1 of the 12-item Short-Form Survey (SF-12), which uses a 5-point Likert scale. SF-12 is a multi-language validated measure of self-reported outcomes that assesses an individual’s health. The SRH variable was developed based on the following question to participants: “Overall, do you think your health is (1) “excellent,” (2) “very good,” (3) “good,” (4) “fair,” or (5) “poor”?” Responses to this question were recoded by combining (3), (4) and (5) as one response option, coded as 0, and (1) and (2) as another option, coded as 1. The choice was made to group the first and second answers to emphasize positive options as coding schemes emphasizing negative ratings have been estimated to less accurately reflect an individual’s overall health than this dichotomization [32].

#### Independent Variables

**FIGURE 1:**
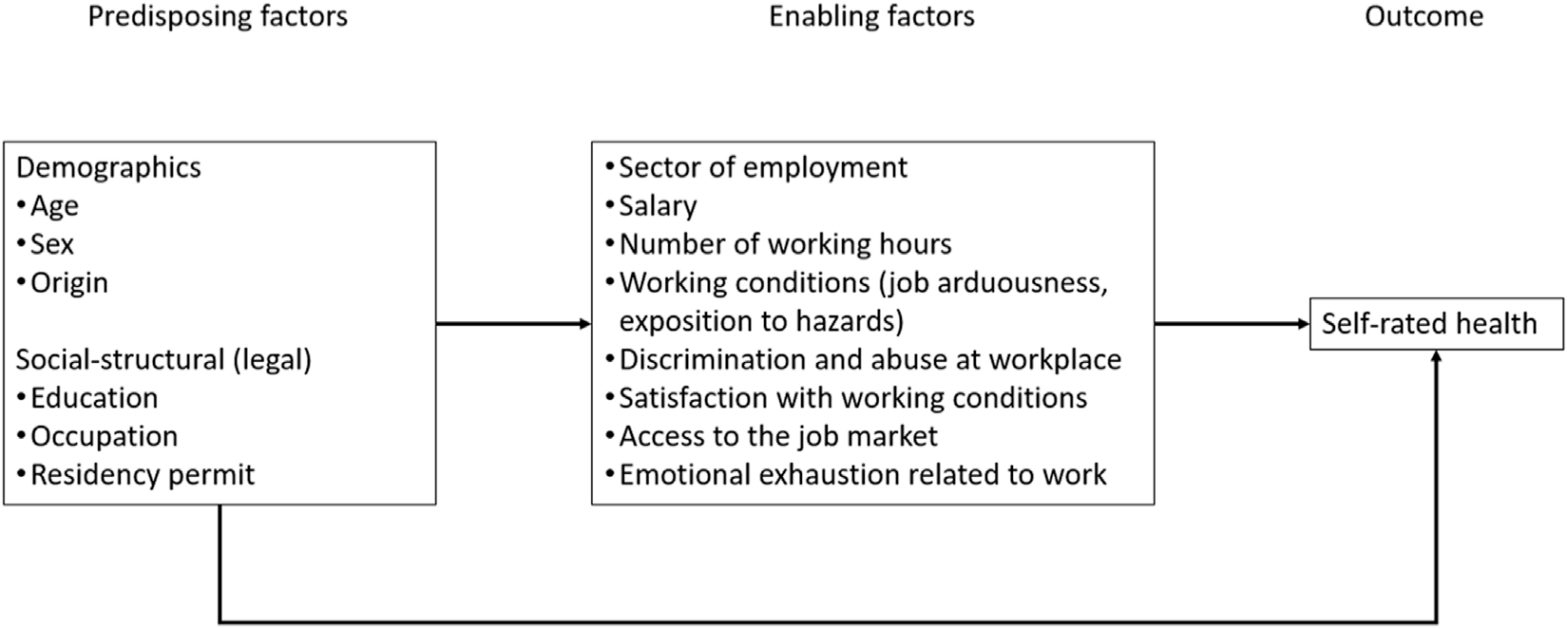
Andersen-Newman framework on self-rated health (Parchemins study, Geneva, Switzerland, 2017–2022).

To assess the influence of work on self-rated health in a broad perspective, we selected a set of indicators that covered three different dimensions: the contractual terms of employment, the working conditions and the satisfaction with working conditions. Figure 1 provides an overview of this approach using the Andersen-Newman framework [33]. Within this framework, we anticipate two main sets of factors influencing the level of self-rated health. On one hand, we expect direct effects stemming from socio-demographic factors. Simultaneously, we anticipate work-related intermediary factors, which typically encompass three dimensions, that could potentially impact self-rated health. For instance, when examining undocumented and recently regularized migrants, this implies the background as working migrant, coupled with the absence of a residence permit, may result in employment that involves more hazards or low wages. These work-related conditions can subsequently influence self-rated health. Since individuals may be subject to several of these factors, our initial approach involves separate testing to identify which factors are relevant to the population under study.

As first dimension we assessed contractual terms. Contractual terms were evaluated through the number of hours of paid work per week, as research has shown that long working hours have a negative impact on health [21]. We categorized hours spent in paid employment into three categories: <29 h per week; between 30 and 45 h per week; and >45 h per week. Similarly, monthly income [in Swiss francs (CHF); CHF 1 = ± Euro 1 and US$ 1] was measured since a lower income is associated with a higher risk of depressive symptoms and anxiety disorders and this relationship is considered as an important determinant of poor SRH [34]. We divided the salary into four categories: < CHF 1,500; CHF 1,500–2,499; CHF 2,500—3,499; and CHF ≥3,500. In Switzerland, the median monthly income was CHF 6,665 in 2020. For the purposes of our analyses, the employment sector (domestic versus others) was also included as an independent variable.

The second dimension assessed working conditions, which embedded the job difficulty and exposure to hazards. Four variables were used: frequency of exposure to painful or tiring positions; the need to carry heavy loads; the requirement to stand upright while working; and the need for repetitive hand or arm movements. Possible responses to each question were “never,” “rarely,” “often” or “almost all the time.” Responses were then combined into a score ranging from 0 to 12 (the option “never” was coded as 0). Results were then categorized into three groups: scores from 0 to 4, “not difficult”; scores from 5 to 7, “difficult” and scores from 8 to 12, “very difficult.” To assess interdependence in the construction of variables, Cronbach’s alpha, a statistical measure assessing the internal consistency of a set of related questions in a questionnaire, was used. Cronbach’s alpha for these four items was 0.59. Although this value is relatively low, this is quite common for this field. Other studies related to this topic have shown similar Cronbach alpha levels [35]. Based on existing findings, we hypothesized that individuals with more difficult working conditions were at greater risk of poorer SRH.

In addition, we measured the frequency of exposure to occupational hazards on the basis of six questions covering the frequency of exposure to vibrations, loud noise, high or low temperatures, passive smoking, and harmful substances in the workplace. The response categories for these questions were again “never,” “rarely,” “often,” or “almost all the time.” The combined results gave a score ranging from 0 to 18, then divided into three categories: We regrouped scores into three categories, with a score 0 to 6 defined as “not exposed,” 7 to 11 as “exposed,” and 12 to 18 as “very exposed.” Cronbach’s alpha for these six items was 0.68.

The third dimension used to assess the influence of work on self-rated health included burnout, satisfaction with working conditions and abuse and discrimination in the workplace. Burnout is a state of physical and psychological fatigue resulting from continuous work-related stress. Maslach developed a validated questionnaire using three dimensions to assess burnout: emotional exhaustion; depersonalization; and loss of sense of personal accomplishment [36]. Occupational mental health was evaluated using the emotional exhaustion dimension of Maslach’s Burnout Inventory (MBI) test. We expected to observe a decreased SRH in the case of high levels of burnout. This dimension was assessed by asking nine questions (alpha = 0.88), such as “do you feel emotionally exhausted by your work,” or “do you feel that you work too hard in your job.” Items were rated on a 7-point frequency scale (0–6), with a total score ranging from 0 to 54 points. A higher score on the emotional exhaustion question indicated a higher likelihood of burnout. In the 4th edition of the MBI manual (2016), Maslach et al. suggested to remove cut-off scores due to a lack of diagnostic validity and argued for treating burnout as continuous data. As such, we did not recode this variable [37].

Satisfaction with working conditions was included as it has been shown to be an important intermediate determinant of mental wellbeing, specifically related to the current work situation. Studies have found that satisfaction with working conditions is positively related to SRH [38]. Thus, we assessed this element by asking respondents to score their satisfaction with their current working conditions on a 0 to 10-point scale, with 0 indicating “complete dissatisfaction” and 10 “complete satisfaction.”

Abuse and discrimination in the workplace were also assessed as previous studies showed that they impacted health, similar to job satisfaction [34]. Participants were asked about exposure to discrimination and abuse related to age, sex, ethnicity or disability at the workplace. The variable was then dichotomized, based on the presence of at least one positive response to the concerned variables.

In addition, access to the job market was included as it may be a source of anxiety that negatively influences the SRH of individuals with limited access. This variable was measured by assessing the difficulty of finding a new job if the current one was lost. Interviewees were asked: “If you lose your current position, would you find a comparable new position?” Possible answers were: “very easily,” “easily,” “difficult,” or “very difficult.” The “very easy” and “easy” modalities were recoded together, and the “difficult” and “very difficult” modalities were combined.

A dichotomous variable measuring eligibility for regularization was created to consider the influence of residence status. Newly regularized migrants and those who had already submitted an application for regularization were grouped into the category “regularized.” Undocumented migrants who had not applied for regularization or were not eligible for “Operation Papyrus” represented the “undocumented group” (control group). This distinction reflected the real situation as all applicants who fulfilled the full set of criteria for regularization and had submitted an application were granted a residence permit within the context of “Operation Papyrus.” By contrast, those who had considered submitting an application, but failed to meet all criteria for acceptance, were advised by the State Administration and migrant support groups not to apply, given the risk of deportation in the case of refusal.

Sex, age, and origin were used as control variables as they potentially influence SRH.

#### Statistical Analysis

Categorical variables were presented as absolute numbers and proportions. Continuous variables were presented as medians and interquartile ranges (IQR). We first performed bivariate analyses and the chi-square test or *t*-test according to the type of variable and between each variable and SRH. Significance was established at a *p*-value < 0.05. Factors with a *p*-value > 0.2 were not included in the multivariable analysis, except for the control variables that were non-significant. We then performed a multivariable logistic regression to further analyze the strength and direction of the association between SRH and the independent variables. The model contained variables related to working conditions, regularization, and controls for age, sex, and origin. The significance level for the regression was set at *p* < 0.05 and results were displayed as adjusted odds ratios (aOR) and 95% confidence intervals (CI). Stata was used to perform all analyses.

## Results

Of the 464 participants interviewed at baseline, we included 395 (85.1%) respondents in our analysis and excluded 69, either because of missing values (*n* = 48) or because they were unemployed (*n* = 21). Table 1 shows the sociodemographic characteristics and work-related factors of study participants stratified by SRH. Overall, participants were mainly female (75.7%) [median age: 43 years (IQR: 15 years)] from Latin America (67.1%), and 204 (51.6%) were undocumented. A total of 147 (37.2%) participants reported “very good to excellent” health. Most were employed in the domestic sector (77.2%). Almost one-half (46.1%) earned less than CHF 2,500 per month and 61% worked 30 or more hours per week. The median score for satisfaction with working conditions was 7 out of 10 points (IQR = 4). While a large majority (80.3%) rated their work as “difficult” or “very difficult,” the mean score of the Maslach scale was 8 out of 54 points (IQR = 14) and only 11.6% reported being exposed to occupational hazards “frequently or very frequently.” Taken together, 21.4% reported exposure to abuse or discrimination at the workplace, and 58.5% indicated having problems finding new work opportunities after losing a job.

**TABLE 1:** Work-related factors and sociodemographic characteristics among study participants stratified by self-rated health (Parchemins study, Geneva, Switzerland, 2017–2022).

Independent variables	Self-rated health			
	Poor to good	Very good to excellent	Total	*p*-value
	*N* = 248 (62.8)	*N* = 147 (37.2)	*N* = 395 (100)	
	*n* (%) or median (IQR)	*n* (%) or median (IQR)	*n* (%) or median (IQR)	
Salary per month (CHF)				<0.001
<1,500	55 (22.2)	20 (13.6)	75 (19.0)	
1,500–2,499	78 (31.5)	29 (19.7)	107 (27.1)	
2,500–3,499	77 (31.0)	53 (36.1)	130 (32.9)	
≥3,500	38 (15.3)	45 (30.6)	83 (21.0)	
Number of weekly working hours				0.066
≤29	106 (42.7)	48 (32.7)	154 (39.0)	
30–45	107 (43.2)	81 (55.1)	188 (47.6)	
≥46	35 (14.1)	18 (12.2)	53 (13.4)	
Job difficulty				0.017
Not difficult	39 (15.7)	39 (26.5)	78 (19.7)	
Difficult	115 (46.4)	67 (45.6)	182 (46.1)	
Very difficult	94 (37.9)	41 (27.9)	135 (34.2)	
Exposure to hazards				0.906
Not exposed	220 (89.1)	127 (87.6)	367 (88.4)	
Exposed	24 (9.7)	16 (11.0)	40 (10.4)	
Very exposed	3 (1.2)	2 (1.38)	5 (1.2)	
Maslach emotional exhaustion score	9 (16)	5 (13.6)	8 (14)	<0.001
Access to the job market				<0.001
Easy	84 (33.9)	80 (54.4)	164 (41.5)	
Difficult	164 (66.1)	67 (45.6)	231 (58.5)	
Discrimination and abuse at workplace				0.379
Never experienced	186 (77.2)	119 (80.9)	305 (78.6)	
Has experienced	55 (22.8)	28 (19.1)	83 (21.4)	
Sector of employment				0.035
Domestic work	200 (80.6)	105 (71.4)	305 (77.2)	
Others	48 (19.4)	42 (28.6)	90 (22.8)	
Satisfaction with employment conditions	7 (3)	8 (2)	7 (4)	<0.001
Residency status				0.001
Undocumented	144 (58.1)	60 (40.8)	204 (51.6)	
Regularized	104 (41.9)	87 (59.2)	191 (48.4)	
Sex				0.128
Female	194 (78.2)	105 (71.4)	299 (75.7)	
Male	54 (21.8)	42 (28.6)	96 (24.3)	
Age (years)	43 (16)	43 (15)	43 (15)	0.605
Origin				0.001
Latin America	179 (72.2)	86 (58.5)	265 (67.1)	
Africa	13 (5.2)	3 (2.0)	16 (4.05)	
Asia	45 (18.2)	39 (26.5)	84 (21.3)	
Europe	11 (4.4)	19 (12.9)	30 (7.6)	

*n* = 395; IQR, interquartile range; CHF, Swiss francs. Data source: Parchemins study.

In bivariate analyses, significant associations were found between a better SRH and origin (*p* < 0.001), having a legal status (*p* < 0.001), higher salary (*p* < 0.001), lower job difficulty (*p* = 0.017), better access to the job market (*p* < 0.001), higher satisfaction with working conditions (*p* < 0.001), and working outside of the domestic sector (*p* = 0.035). No association was found between SRH and exposure to occupational hazards, the level of emotional exhaustion, and exposure to abuse or discrimination at the workplace.

Table 2 shows the results of the multivariable regression analysis to identify factors associated with SRH. The model showed that East Asian origin (aOR = 1.89; 95% CI = 1.08–3.30) and satisfaction with working conditions (aOR = 1.16; 95% CI = 1.04–1.31) were positively associated with better SRH. By contrast, workers performing very difficult tasks (aOR = 0.47; 95% CI = 0.24–0.94) and having more difficulties finding a new job (aOR = 0.60; 95% CI = 0.37–0.98) were less likely to describe their health as “very good” or “excellent.” The association between SRH and income, the number of paid working hours, and the level of emotional exhaustion were not statistically significant. Legal status regularization showed a trend toward a significant association with better SRH (aOR = 1.54; 95% CI = 0.95–2.50; *p* = 0.078). The model presents a McFadden’s pseudo-*R* ^2^ of 0.115, which is considered satisfactory.

**TABLE 2:** Multivariable associations between better self-rated health and work-related factors and sociodemographic characteristics (Parchemins study, Geneva, Switzerland, 2017–2022).

Variables (reference)	Self-rated health	
	aOR (95% CI)	*p*-value
Number of weekly working hours (≤29)		
30–45	0.89 (0.49–1.66)	0.734
≥46	0.93 (0.39–2.20)	0.865
Salary (<1,500 CHF)		
1,500–2,499	0.82 (0.39–1.72)	0.596
2,500–3,499	1.25 (0.55–2.84)	0.586
≥3,500	2.11 (0.83–5.69)	0.116
Sector of employment (domestic work)		
Other	1.36 (0.60–3.10)	0.466
Job difficulty (Not difficult)		
Difficult	0.56 (0.31, 1.04)	0.065
Very difficult	0.47 (0.24–0.94)	0.032
Maslach—Emotional exhaustion	0.99 (0.97–1.02)	0.621
Access to the job market (Easy)		
Difficult	0.60 (0.37–0.98)	0.040
Satisfaction employment conditions	1.16 (1.03–1.30)	0.016
Status (Undocumented)		
Regularized	1.54 (0.95–2.50)	0.078
Sex (Male)		
Female	1.15 (0.53–2.48)	0.718
Age, years	1.00 (0.98–1.03)	0.828
Origin (Latin America)		
Africa	0.46 (0.12–1.82)	0.271
Asia	1.89 (1.08–3.30)	0.025
Europe	1.64 (0.60–4.54)	0.335
AIC	497.24	
Pseudo-*R* ^2^	0.115	

aOR, adjusted odds ratio; CI, confidence interval; CHF, swiss francs; AIC, Akaike Information Criterion; Data source: Parchemins study.

## Discussion

The aim of this study was to determine the relationship between working conditions and self-rated health among undocumented migrants and those engaged in the process of regularization in Switzerland. Our findings highlight the context in which such migrants live and work and showed that migrants’ origin, the difficulty of their work, the satisfaction with their working conditions, and their access to the job market significantly influenced SRH. However, we only found a borderline association between obtaining a residency permit and better health. This is in line with earlier findings where the effects of regularization were outweighed by other socioeconomic factors [9].

Consistent with our observations, previous studies on physical and psychosocial conditions have shown that the physical difficulty of working conditions negatively affected SRH. Psychosocial factors, such as those measured with the MBI score, may frequently result in little or no effect on this health indicator, even for those working and living in precarious conditions [39]. Likewise, the difficulty in accessing the job market can be explained by several factors, including the need to remain as “invisible” as possible to avoid denunciation. Most migrant workers seek jobs in the informal economy where employers can exploit their limited bargaining power and thus impose exhausting working conditions.

Interestingly, our findings showed that while salaries are well below legal minimum wages in the informal sector compared to the regular sector in Switzerland, only a minority of workers reported abuse or discrimination. While one could assume that harsh working conditions would lead to a higher level of reported abuse or discrimination, the relation is not significant. This may result from a comparison with salary levels in the home country, the financial necessity to make a living in a city with a very high cost of living, and also from the internalization of the misbalance of power between migrants in irregular situation and their employers as previously shown in Spain [21]. Of note, eligibility to apply for regularization requires generating a sufficient income and migrant workers’ frequent lack of legal rights to oppose the risk of dismissal at any time may also contribute to the acceptance of unfair contractual conditions of employment. The association between satisfaction with working conditions and SRH is consistent with the results found by Pikhart et al. [40] who reported the positive effect of satisfaction with employment conditions on the SRH of undocumented immigrants in the Czech Republic.

No relationship between exposure to occupational hazards and SRH was identified in our study. Similar to a previous investigation of immigrant workers’ perceptions of working conditions in Spain, a majority of our sample were women working in the domestic sector, suggesting that they regularly performed similar tasks in their paid jobs as in their homes, thus potentially diminishing the perception of risk. This would explain the null finding of a relationship between occupational exposure and SRH [21]. By contrast, according to Buchmüller et al., consumer knowledge about the potential risks of household chemicals is generally low and many of the domestic workers in our sample may not be aware of the impact of product exposure on their health [41]. Our research has a particular focus on SRH, a subjective measure that has been successful in assessing physical morbidity and mortality. However, this measure does not reflect objective health status and our results, suggesting that exposure to hazards does not decrease SRH, do not imply that it does not negatively affect the objective health status of the population studied. Indeed, as mentioned, prior studies have observed that exposure to toxic products and dust among household cleaners was significantly associated with an increased risk of developing respiratory problems, including asthma [23].

Similar to Sousa et al. [14], our study showed that SRH was more affected by working conditions than by legal status. A possible explanation may be that data collection occurred very early during the regularization process. Thus, changes in living conditions that can have a potential impact on SRH may not have had the time to fully evolve. In addition, while migrant workers may enjoy positive changes in their living conditions after regularization, they may remain in a position of financial vulnerability as this transition is associated with new material and immaterial sources of stress, including payment of taxes and compulsory health insurance, as well as compliance with the conditions for renewal of the residence permit [42]. These concerns could have a significant impact on mental health, leading to a mitigation effect in the evaluation of their own health. Of note, the *Parchemins* study and other similar research projects in Europe have shown how undocumented migrants tended to disconnect the subjective evaluation of their own health from the objective measurement of mental health suffering [9, 11, 12]. Despite not observing the early effects of regularization on SRH, regularization nevertheless confers new labor rights and leverage for migrants to improve their working conditions and terms of employment, including the ability to report work-related injustices without fearing deportation. For these workers, regularization empowers them to move out of the informal economy and expand their employment opportunities, especially for occupations that better match their skills and qualifications prior to their migration. As a result, this endows them with a greater degree of control over their own lives. Further studies are clearly needed to assess the long-term impact of regularization on working conditions and SRH.

Our findings differ from previous studies reporting on factors influencing self-rated health in migrant workers. For instance, Vianello et al. found an association between SRH and work-related abuse and discrimination among migrant females working in the domestic sector in Italy using qualitative methods [43]. Similarly, Pikhard et al. observed that the risk of reporting poor SRH was higher among females in the Czech Republic [40]. This highlights the variability of findings about undocumented migrants whose health, working and living conditions are heavily influenced by local political, social and economic contexts. It calls for caution in generalizing evidence about this highly heterogeneous population.

Our study has several limitations. First, the cross-sectional nature of the data reduces the ability to assess for causality in the observed associations as it does not consider changes over time in the SRH status of the studied population, which can rapidly evolve [44]. Second, we analyzed results from the first wave of the longitudinal *Parchemins* study, thus making it difficult to evaluate not only the full impact of regularization on health, but also its influence on work conditions due to the short time since regularization. Third, we observed contradictory responses from several participants throughout data collection. For example, some described difficult working conditions and yet, at the same time, reported a high satisfaction with these same conditions. Fourth, the variables used to measure the difficulty of the job and exposure to hazards had a Cronbach’s alpha coefficient of 0.59 and 0.68, respectively, which are acceptable, but not excellent. The reasons for these relatively low alphas are related to the low correlations between some items and the limited number of items in the scale of difficulty of the job. In order to broadly cover the different aspects of each dimension, scales (rather than individual items) were chosen to measure these phenomena. Finally, our sampling was purposive and therefore cannot claim to be fully representative of the source population in Geneva.

Despite these limitations, the present study contributes new and important insights into the conditions that migrant workers are grappling with, an invisible population that is particularly hard to reach. The sample is relatively unique in a field of study where quantitative research is scarce and such studies help to strengthen the knowledge base about the occupational health of undocumented and recently regularized migrants.

### Conclusion

Work-related factors influenced self-rated health to a greater extent than legal status regularization in migrant workers in the early phase of the regularization policy implementation. The fact that undocumented and newly regularized migrant workers were exposed to occupational factors negatively affecting their health highlights the need for targeted policies aiming to ensure safer and better contractual terms of employment and working conditions, especially in sectors that are insufficiently unregulated. Considering the difficulties to access to the labor market and to generate a sufficient income, policies should also ensure access to financial, material and food support, even more so in a period of instability such as during the COVID-19 pandemic.
